# Systematic Assessment of Nonproteolytic Clostridium botulinum Spores for Heat Resistance

**DOI:** 10.1128/AEM.01737-16

**Published:** 2016-09-16

**Authors:** Ewelina Wachnicka, Sandra C. Stringer, Gary C. Barker, Michael W. Peck

**Affiliations:** Institute of Food Research, Norwich Research Park, Colney, United Kingdom; Rutgers, The State University of New Jersey

## Abstract

Heat treatment is an important controlling factor that, in combination with other hurdles (e.g., pH, a_w_), is used to reduce numbers and prevent the growth of and associated neurotoxin formation by nonproteolytic C. botulinum in chilled foods. It is generally agreed that a heating process that reduces the spore concentration by a factor of 10^6^ is an acceptable barrier in relation to this hazard. The purposes of the present study were to review the available data relating to heat resistance properties of nonproteolytic C. botulinum spores and to obtain an appropriate representation of parameter values suitable for use in quantitative microbial risk assessment. In total, 753 *D* values and 436 *z* values were extracted from the literature and reveal significant differences in spore heat resistance properties, particularly those corresponding to recovery in the presence or absence of lysozyme. A total of 503 *D* and 338 *z* values collected for heating temperatures at or below 83°C were used to obtain a probability distribution representing variability in spore heat resistance for strains recovered in media that did not contain lysozyme.

**IMPORTANCE** In total, 753 *D* values and 436 *z* values extracted from literature sources reveal significant differences in spore heat resistance properties. On the basis of collected data, two *z* values have been identified, *z* = 7°C and *z* = 9°C, for spores recovered without and with lysozyme, respectively. The findings support the use of heat treatment at 90°C for 10 min to reduce the spore concentration by a factor of 10^6^, providing that lysozyme is not present during recovery. This study indicates that greater heat treatment is required for food products containing lysozyme, and this might require consideration of alternative recommendation/guidance. In addition, the data set has been used to test hypotheses regarding the dependence of spore heat resistance on the toxin type and strain, on the heating technique used, and on the method of *D* value determination used.

## INTRODUCTION

Over the last few decades, there has been an increasing demand from consumers in many countries for innovative, ready-to-eat, minimally processed chilled foods such as complete meals, prepared salads and vegetables, pizza, sandwiches, sliced cooked meat, soups, and cakes (1, 2). These products have many desirable properties, such as improved taste, high nutritional value, convenience, and minimal use of additives and preservatives, but simultaneously, they also present challenges with regard to food safety. In the United Kingdom, for example, the microbiological safety of many products relies on a combination of moderate heat treatment (generally in the range of 70 to 90°C), chilled storage (at 8°C or below), a restricted shelf life (typically, a maximum of 42 days), good-quality raw materials, and hygienic manufacturing in the absence of added preservatives (1–3).

Strains of Clostridium botulinum form the highly potent botulinum neurotoxin, and consumption of food containing as little as 30 ng of preformed toxin can cause botulism, a severe paralytic disease (3–6). C. botulinum is a heterogeneous bacterial species that can be separated into four discrete groups, with strains in two groups, proteolytic C. botulinum (group I) and nonproteolytic C. botulinum (group II), being responsible for food-borne botulism (3, 5, 6). Proteolytic C. botulinum has a minimum growth temperature of 10 to 12°C (6) and is a concern for chilled foods following significant temperature abuse. Hazards arising from nonproteolytic C. botulinum are a particular concern for the safety of minimally processed chilled foods, because not only can spores of this pathogen survive mild heat treatments, but they can germinate, giving cells that multiply and form neurotoxin at 3.0 to 3.3°C in 5 to 7 weeks (6, 7). Strains of nonproteolytic C. botulinum produce a single neurotoxin of type B, E, or F, with incomplete neurotoxin gene fragments also reported (8, 9). Mass-produced, minimally processed chilled foods have a very strong safety record, but there have been occasional incidents, most frequently involving type B or E neurotoxin, that have been associated with time and/or temperature abuse during storage or with various home-prepared foods (1, 2, 10–14). The estimated cost of each food-borne botulism case in the United States is ∼$30 million (15).

Considering the increasing consumer demand for chilled foods, the potentially severe adverse health effects, and the serious commercial implications of food-borne botulism, the control of nonproteolytic C. botulinum in minimally processed chilled foods is essential. Identification and optimization of appropriate controls (e.g., effective heating time), based on sound science, as well as their consistent application and a systematic approach to inform the public concerning safety, are a priority. One approach is quantitative microbiological risk assessment (QMRA), which combines appropriate elements together in an accessible framework to calculate the associated risk (16, 17). This approach requires robust scientific data, for example, on the spore loading in raw materials (18, 19) and on spore heat resistance. In this study, we gathered and analyzed evidence that relates to the heat resistance of nonproteolytic C. botulinum spores and established a representation that can be useful in the development of full risk assessments. An appropriate consideration of uncertainty is essential in both analysis and communication, so that outcomes of this investigation are presented in terms of probability to represent quantified beliefs.

Seven other reviews concerning the heat resistance of spores of nonproteolytic C. botulinum can be identified (20–26). These studies vary in their approaches and in their coverage of heating regimens but are a valuable source of information about factors influencing spore heat resistance (e.g., the type of strain, the composition of the heating menstruum, and/or recovery conditions), as well as indicate important heterogeneity and a range of *D* and *z* values for nonproteolytic C. botulinum in different heating menstrua at different temperatures. The review by van Asselt and Zwietering (26) is a valuable and extensive collection of information relating to the heat resistance of many food-borne pathogens, but in the case of spores of nonproteolytic C. botulinum, the chosen reference temperature, *T* = 120°C, is very high so that it is difficult to translate the information to practical food-manufacturing processes.

We have gathered and organized data to derive probabilistic distributions for parameters of spore heat resistance models for inclusion in QMRA and other food safety quantifications that have direct relevance to food manufacture. A complementary meta-analysis was recently done by Diao et al. (27) to express the heterogeneity observed in the heat resistance properties of spores of proteolytic C. botulinum at high temperatures.

## MATERIALS AND METHODS

### Population kinetics.

A simple picture of a homogeneous spore population identifies *N*_0_ spores acting individually but each with the same properties. In this case, the population kinetics, in the presence of isothermal heat inactivation, follows a log-linear relationship so that *N*(*t*) = *N*_0_10^−*t*/*D*(*T*)^, where *N*(*t*) is the size of the population after time *t* and *D*(*T*) is the decimal reduction time (*D* value) at temperature *T*. An estimate of the *D* value of a population can be obtained from a log-linear plot of the population size against time. In turn, the temperature dependence of the *D* value is usually related to a simple temperature difference and a single parameter, *z*, D(T_2_) = D(T_1_)10^(T_1_^ ^− T_2_^^)^^/*z*^, which assigns an appropriate temperature scale, i.e., the temperature difference that makes the *D* value change by a factor of 10 (this parameter is usually known as the *z* value). This relationship ensures that a *D* value measured at one temperature, *T*_1_, can be used to predict the heat resistance at a second temperature, *T*_2_. Many alternative forms of population kinetics have been considered in relation to the thermal inactivation of bacterial populations (e.g., see reference 28), but in a consideration of large quantities of data from many distinct sources, the two relationships above, defining the *D* and *z* values, are the most general and therefore the most appropriate. An important exception to the simple behavior above describes a single population composed of two different subpopulations. Assuming that the *D* values of the two subpopulations are sufficiently distinct, the population kinetics will appear as two separate line segments on a log-linear plot and it is practical to estimate two *D* values (one for each subpopulation).

### Literature review.

Information relating to *D* and *z* values for spores of nonproteolytic C. botulinum was collected following searches of electronic databases, scientific journals, books, and technical reports in a manner similar to that described previously (18). Searches of electronic databases were conducted by using keywords that included botulinum, spores, heat, temperature, inactivation, thermal, and survival. Searches were not restricted by country or language, and non-peer-reviewed articles were included. The references of the identified articles and relevant review articles were searched for potential omissions. The information reviewed includes studies published before 2011.

Each of the identified sources was assessed against the following 10 criteria for inclusion prior to data extraction:

1. The major aim of the study was to measure heat resistance of nonproteolytic C. botulinum spores.
2. The thermal treatment was performed with a wet process but did not include high-pressure processing, a pulsed electric field process, or microwave energy.
3. The experiments were isothermal in a temperature range of 50 to 95°C.
4. The bacterial strain name or the toxin type was identified.
5. The heating menstruum and the heating method were adequately described.
6. The recovery method, media, and conditions were adequately described.
7. The *D* and *z* values were provided in the report, or it was possible to calculate them from data.
8. The period of inactivation used to determine the *D* value covers more than 1 order of magnitude in the population size.
9. No inhibitory factors (e.g., a low pH of <4.1) are included in the heating menstruum or during recovery.
10. Kinetic data clearly separate a heat-sensitive and a heat-resistant fraction for systems that include lysozyme in the recovery medium (see below for further details).

When the *D* value was not reported explicitly but the source provided a temperature at which the heat treatment was performed, the starting inoculum size, and the number of surviving spores, the *D* value was determined from tables or figures by fitting the best straight line. When the experiments were conducted by using a TDT (thermal death time) method that determines the time it takes to inactivate a population of spores, the *D* value was calculated as $\sqrt{\left(t_{\max}\;t_{\min}\right)}/\log N_{0}$, where *t*_max_ is the longest heating time for which spores could be recovered, *t*_min_ is the shortest time for which no recovery was possible, and *N*_0_ is the initial number of microorganisms (e.g., see reference 29). Some *z* values were determined from reported or calculated *D* values.

Extracted data were labeled by strain and toxin types, by the heating menstruum, by the heat treatment temperature, by the *D* value (in minutes), by the *z* value (in degrees Celsius), and by the method of calculation. Additionally, each record includes notes on the heating method, the recovery medium, and the experimental conditions. Where possible, the pH, water activity, and nutrients present in the heating menstruum were also recorded. All data records are included in the supplemental material.

Previous publications indicated that the presence of lytic enzymes (notably lysozyme) during recovery has a large effect on the measured *D* values for spores of nonproteolytic C. botulinum (30–41). Therefore, the collected data are segmented into two major subsets; −LYS (recovery of spores in the absence of lysozyme) and +LYS (recovery of spores in the presence of lysozyme). Sometimes lysozyme is deliberately added to the recovery medium, but in other cases, both heat treatment and recovery are conducted in a food substrate where the activity of lytic enzymes has previously been observed (e.g., hen egg white, seafood, vegetables). In the presence of lysozyme, survival curves are generally biphasic (indicating two subpopulations of spores), and a *D* value is assigned to each part of the curve, i.e., for each subpopulation of spores (a heat-sensitive subpopulation, +LYS HS, whose spore coats are impermeable to lysozyme and a heat-resistant subpopulation, +LYS HR, whose spore coats are permeable to lysozyme). In this case, spore heat resistance values were only included for kinetic data that clearly separated the heat-sensitive and -resistant fractions.

A majority of the tests were carried out with single strains of nonproteolytic C. botulinum; however, some experiments relate to mixtures of strains, often of different toxin types (i.e., cocktails), and are referred to as mixed-strain experiments.

### Representing uncertainty.

For a particular population of spores under static conditions, the *D* value is fixed but uncertain. In principle, the *D* value can have any nonnegative value, and in some cases, reported values cover a very large range so that a log-normal distribution is a natural representation of the uncertainty, *p*[*D*(*T*)] ∼ lognormal(μ, σ), where μ and σ are the mean and standard deviation of ln[*D*(*T*)] [the parameters are easily transformed to other statistical descriptors such as the mean and standard deviation of *D*(*T*)].

The *z* value is also fixed but uncertain. It is practical to assume that the *z* value is bounded and has quite a restricted range. A four-parameter beta distribution is suitable to represent this uncertainty (e.g., see reference 42) as *p*(*z*) ∼ *Beta*(*a*, *c*, α_1_, α_2_), where *a* and *c* are the limits of the range of *z* values and α_1_ and α_2_ are exponents that determine the shape of the distribution. The mean value, <*z*>, is given in terms of the parameters as follows: <*z*> = *a* + [α_1_/(α_1_ + α_2_)](*c* − *a*).

Distribution parameters for *D*(*T*) and *z* values can be obtained by simple least-squares fitting of the appropriate cumulative distribution to the empirical distribution of the data (e.g., with the add-in package Solver for Microsoft Excel [2010]). For the *z* value, the fitted parameters are subject to some constraints, such as, e.g., that α_1_ and α_2_ are >0, to ensure that the distribution is unimodal. Upper and lower confidence intervals for the parameter estimates, and the assessment of goodness of fit, can be calculated according to a method described by Brown (43).

The aggregated data describing *D* values reflect measurements at several temperatures, but since the values at different temperatures are connected by the *z* value, it is possible to capture this variation within a single representation at a fixed reference temperature. In this case, we use *T* = 80°C as a reference since it is the dominant temperature for data collection (when lysozyme is absent during recovery). Combining complex information into a single representation of thermal inactivation of nonproteolytic C. botulinum is a valuable step toward the consistent application of safety in food manufacturing environments.

The process of temperature transformation for decimal reduction time is extended to include probabilistic information about *D*(*T*) and *z* (in the expression above, *T*_1_ is the experimental temperature and *T*_2_ is the reference temperature). A distribution for *D*′(80) is obtained by direct integration of the expression that describes the transformation of *D* values or, more simply, by a Monte Carlo process that involves sampling a distribution of *D* values measured at *T*_1_, *p*[D(*T*_1_)], and a distribution of *z*, *p*(*z*), repeatedly, e.g., with @RISK software version 5.5.1 (Palisade, USA)—the parent distributions are fitted to the experimental data. A notation *D*′(80) is used to indicate a *D* value that was measured at one temperature and then expressed at 80°C to discriminate it from direct measurements. A complete distribution for *D*′(80), based on the full data set, can be built as a weighted sum of distributions obtained from data at distinct experimental temperatures (weights are determined by the number of *D* value determinations at one particular temperature).

### ANOVA.

Statistical analyses were used to identify factors that influence the magnitude of parameters describing the probability distributions for *D* and *z* values. Differences between groups of logarithmically transformed *D* values converted to a heating temperature of 80°C, log*D*′(80), were examined by a standard *t* test and, where necessary, by one-way analysis of variance (ANOVA) with SPSS Statistics software version 21 (IBM, USA). Where given, the *z* value corresponding to the same set of conditions was used; otherwise, the value of <*z*> estimated in this study for strains of the appropriate toxin type was used.

Two factors investigated in detail were the dependence of spore heat resistance on the toxin type (by pairwise comparisons) and comparison of the heat resistances of the +LYS HS and +LYS HR spore fractions. A comparison of *D* values for the −LYS and +LYS HS fractions was also included. Additionally, the significance of the heating menstruum, heating technique, method of *D* value determination, and strain were examined by pairwise comparison of *D*′(80) in a temperature range of 50 to 83°C. An unsupervised hierarchical clustering approach with a dissimilarity metric based on Euclidean distance was used to indicate a strain classification pattern based on heat resistance properties. Clustering was conducted with SPSS Statistics software.

## RESULTS

### Literature sources.

Searches identified 15,037 titles, of which 46 met all of the inclusion criteria, including unpublished data from the Institute of Food Research and unpublished data from J.-M. Membré, P. McClure. Heat resistance of nonproteolytic C. botulinum spores has been studied in foods, buffer, and media under different recovery conditions over a temperature range of 50 to 95°C. In total, 753 *D* values were extracted from the sources; 253 *D* values correspond to nonproteolytic C. botulinum type B, 375 correspond to nonproteolytic C. botulinum type E, 65 correspond to nonproteolytic C. botulinum type F, and 60 correspond to a mixture of nonproteolytic C. botulinum strains. There were 549 *D* values from −LYS studies (originating from 39 references) and 194 *D* values from +LYS studies (originating from 10 references). For the +LYS studies, the data set includes 89 *D* values for heat-sensitive (HS) subpopulations (measured in a temperature range of 75 to 93°C) and 105 *D* values for heat-resistant (HR) subpopulations (measured in a temperature range of 75 to 95°C). A heating temperature of 80°C was used most frequently for experiments without lysozyme (151 *D* values). Other dominant temperatures for determination of *D* values were 70, 75, 77, 79, and 82°C, corresponding to 59, 66, 51, 37, and 56 data points. When lysozyme was added to the recovery medium, the temperature at which the heat resistance of spores was determined was generally higher; 90°C was dominant (88 data points), followed by 85°C (41 data points), and 80°C (22 data points). In total, 436 *z* values were collected from eligible studies. The majority of the data points were recorded for −LYS studies (366 *z* values). The literature data on spore heat resistance are summarized in the supplemental material.

### Probability distributions of *z* values.

**FIG 1:**
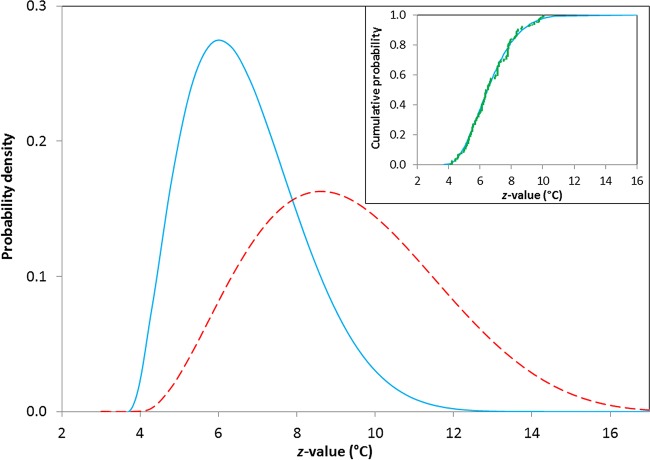
Beliefs concerning *z* values (in degrees Celsius) for spores of nonproteolytic C. botulinum. The solid blue line represents *z* values measured without lysozyme in the recovery media (−LYS), <*z*> = 6.7°C, and the dashed red line represents *z* values measured for the HR fraction with lysozyme in the recovery media (+LYS HR), <*z*> = 9.3°C. In the inset, green squares represent the empirical distribution of the data and the blue line represents the cumulative probability for experiments conducted in the absence of lysozyme in the recovery media at temperatures between 50 and 83°C (*n* = 338).

Probability distributions that represent *z* values from experiments with and without lysozyme are illustrated in Fig. 1. The inset represents the fitted cumulative distribution and the empirical data for *z* values measured in systems without lysozyme. A similar fit is obtained for systems with lysozyme (data not shown). For data collected in the absence of lysozyme (−LYS), the fitted beta distribution (blue solid line) has parameters *a* = 3.7°C (2.9°C, 4.5°C), *c* = 16.5°C (6.0°C, 27.0°C), α_1_ = 2.9 (0.8, 5.0), and α_2_ = 9.2 (0, 24.0), and for data collected in the presence of lysozyme (heat-resistant fraction; +LYS HR), *z* values correspond to beta parameters *a* = 4.0°C (0, 16.4), *c* = 19.6°C (0, 77.0), α_1_ = 3.1 (0, 20.6), and α_2_ = 6.0 (0, 59.0) (values in parentheses are 95% confidence intervals). There are too few data points to merit calculation of the distribution of *z* values for the heat-sensitive fraction (+LYS HS). Fits are obtained subject to the constraint that *c* is greater than or equal to the largest observed value of *z*. The corresponding mean values are <*z*> = 6.7°C (4.4°C, 10.0°C) for −LYS and <*z*> = 9.3°C (5.4°C, 14.3°C) for +LYS HR.

### Probability distributions of *D* values.

**FIG 2:**
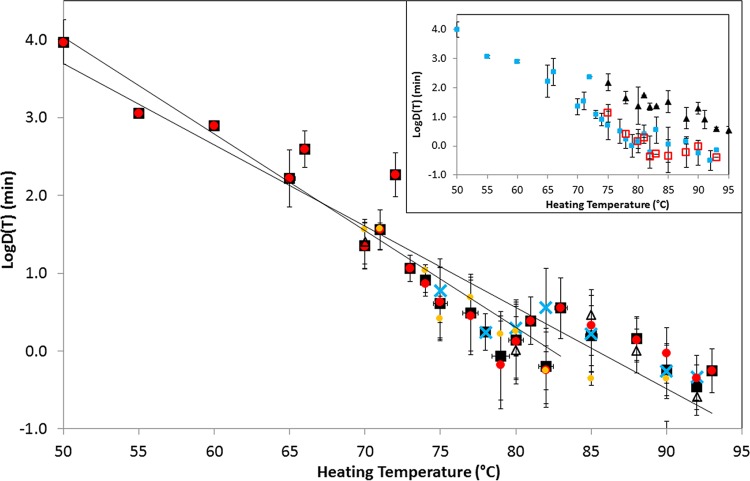
Summary of the heat resistance (*D* values) of spores of nonproteolytic C. botulinum extracted from literature sources. Data points and error bars correspond to the mean and standard deviation of all of the *D* values measured at a particular experimental temperature in the absence of lysozyme. Black squares correspond to all of the data, and blue crosses, red circles, orange circles, and open triangles correspond to toxin types B, E, and F and mixed toxin types. The solid lines represent the best fit to the experimental data in a temperature range of 50 to 83°C and for the whole data set. The inset compares *D* values measured in the absence of lysozyme (blue closed squares) and those measured in the presence of lysozyme (HS fraction, red open squares; HR fraction, closed triangles).

A summary of the data representing *D* values is shown in Fig. 2, where each data point and error bar represents the mean and standard deviation of the logarithm of the *D* values measured at one particular temperature (experimental temperatures were 50 to 93°C, and temperatures measured on the Fahrenheit scale were converted to the nearest whole degree Celsius). The inset in Fig. 2 shows a comparison of the *D* values measured in the absence and presence of lysozyme (HS and HR fractions) in recovery media. The measured spore heat resistance was similar in the absence of lysozyme and for the heat-sensitive (HS) fraction in the presence of lysozyme but was higher for the heat-resistant (HR) fraction in the presence of lysozyme (Fig. 2).

By considering the *D* values obtained in the absence of lysozyme from increasing ranges of experimental temperatures, after transformation to a reference temperature of 80°C, it is possible to identify an important property of the thermal inactivation data set. For data obtained (in the absence of lysozyme) from experiments in temperature ranges of 50 to 79°C, 50 to 80°C, 50 to 81°C, 50 to 82°C, and 50 to 83°C, an Anderson-Darling statistic (*A*^2^) is consistently in the range of 1.4 to 2.2. In contrast, the data obtained from experiments in temperature ranges of 50 to 85°C, 50 to 88°C, 50 to 90°C, and 50 to 93°C have an Anderson-Darling statistic in the range of 4.1 to 5.9. The Anderson-Darling test measures the approximate “normality” of a set of empirical values (e.g., see reference 44), and in this case, the sudden change in the value of *A*^2^ indicates a significant change in the statistics that underpin the variation of *D* values collected at higher temperatures. Taking account of this finding, Fig. 2 shows the line of best fit for *D* values measured in a temperature range of 50 to 83°C and also the line for the entire data set. A similar pattern of temperature dependence is observed for *D* values that correspond to the HS fraction of the +LYS data set but not for *D* values that correspond to the HR fraction of the +LYS data set (*A*^2^ is 1.0 to 2.2 and shows no rise at higher heating temperatures). The Anderson-Darling values do not depend strongly on the *z* value used for transformation of *D* values (where possible, the measured *z* value was used).

### Effect of lysozyme during recovery of heated spores.

The parameters of fitted normal distributions for the decimal logarithm of the *D* values of spores of nonproteolytic C. botulinum are shown in Table 1. The *D* values measured in the absence of lysozyme (−LYS) and for the HS fraction in the presence of lysozyme (+LYS HS) are both significantly smaller than those for the HR fraction of spores in the presence of lysozyme (+LYS HR).

**TABLE 1:** Parameters of fitted normal distributions corresponding to reported values of log[*D*(*T*)] (minutes) for spores of nonproteolytic C. botulinum^*a*^

*T* (°C)	−LYS			+LYS HS			+LYS HR		
	*n*	<Log[*D*(*T*)]>	σ_log_*_D_*	*n*	<Log[*D*(*T*)]>	σ_log_*_D_*	*n*	<Log[*D*(*T*)]>	σ_log_*_D_*
70	59	1.35	0.28						
75	66	0.71	0.49	4	1.12	0.30	5	2.18	0.28
80	151	0.16	0.41	9	0.16	0.26	9	1.37	0.64
85	27	0.04	0.60	18	−0.35	0.57	22	1.52	0.38
90	8	−0.24	0.42	40	−0.03	0.21	44	1.29	0.20

a Values correspond to measurements of the HS and HR fractions of spores in the presence (+LYS) and absence (−LYS) of lysozyme. *n* is the number of reported values.

By using the probabilities of the measured *z* values (Fig. 1), lethality measurements at different temperatures (including those in Table 1) can be transformed to establish belief about *D* values at 80°C. For experiments performed in the absence of lysozyme (and for the heat-sensitive fraction in the presence of lysozyme), data are restricted to temperatures in the range of 50 to 83°C. The parameters for normal distributions of log[*D*′(80)] are summarized in Table 2. Direct comparison of transformed *D* values, *p*[*D*′(80)], with those measured at 80°C, *p*[*D*(80)] (Table 1), shows that the distributions have very similar locations but the distribution of transformed values includes additional spread from uncertainties associated with the *z* value.

**TABLE 2:** Parameters of fitted normal distributions corresponding to values of log[*D*(*T*)] (minutes), transformed to 80°C, for spores of nonproteolytic C. botulinum^*a*^

Parameter	−LYS	+LYS HS	+LYS HR
*n*	503	27	105
<*D*′(80)>	2.23	1.32	245
σ*_D_*_′(80)_	4.44	0.95	423
<Log[*D*′(80)]>	0.00	0.03	2.09
σ_log[*D*′(80)]_	0.55	0.28	0.51

a Values correspond to measurements of the HS (75 to 83°C) and HR (75 to 95°C) fractions of spores in the presence (+LYS) and absence (−LYS; 50 to 83°C) of lysozyme. *n* is the number of reported values.

Recovery of heated spores of nonproteolytic C. botulinum in the presence of lysozyme (or some other lytic enzymes) results in biphasic survival curves, with HS (+LYS HS) and HR (+LYS HR) fractions (Fig. 2 and Tables 1 and 2). Analysis of the collected data indicates that there is no significant difference (*t* test; *P* = 0.07) between *D* values measured in a heating temperature range of 50 to 83°C and those transformed to 80°C [*D*′(80)] for spores recovered in the absence of lysozyme (−LYS) and the HS (+LYS HS) fraction of spores recovered in the presence of lysozyme. With recovery in the presence of lysozyme, it was estimated that, for the collected data, the +LYS HR fraction constitutes approximately 0.02 to 3.1% of the initial spore population. Statistical analysis indicates a significant difference between the measured *D* values of spores from the +LYS HS and +LYS HR fractions (*t* test; *P* = 0.01). There is also a significant difference between the measured *D* values of −LYS spores and the +LYS HR fraction (*t* test, *P* < 10^−6^).

### Effect of toxin type.

**FIG 3:**
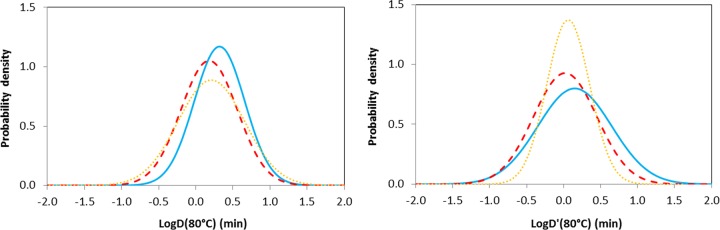
Effect of toxin type on distributions of log[*D*(80)] and log[*D*′(80)] (min) for spores of nonproteolytic C. botulinum recovered in the absence of lysozyme. Isothermal values are measured at 80°C, and the transformed values [*D*′(80)] are converted from data established in a heating temperature range of 50 to 83°C. The blue solid line represents toxin type B strains, the red dashed line represents toxin type E strains, and the orange dotted line represents toxin type F strains.

Analysis (ANOVA and paired *t* tests) of log*D*′(80) values established in the range of *T* = 53 to 83°C in the absence of lysozyme shows that there is a small but significant difference in the spore heat resistance of strains with different toxin types {<log*D*′(80)> = 0.15, 0.02, and 0.06 and σ_log[*D*′(80)]_ = 0.50, 0.43, and 0.29 for types B, E, and F with *P* = 0.03 and <*z*> = 6.9, 6.9, and 6.5°C}; representative normal distributions are indicated in Fig. 3 along with corresponding isothermal distributions. It is difficult to be precise about the origin of this difference, but it may correspond to the inclusion of measurements from the lowest experimental temperatures (the difference disappears if the data are restricted to *T* ≥ 75°C). Strains forming type B toxin more frequently showed higher heat resistance than strains forming type E toxin. For log[D(80)], there is no significant difference (*P* = 0.18) in spore heat resistance for different toxin types. Similarly, there was no significant difference in heat resistance between the toxin types of spores recovered in the presence of lysozyme {<log*D*′(80)> = 2.01, 2.00, and 2.41 and σ_log[*D*′(80)]_ = 0.53, 0.49, and 0.05 for types B, E, and F with *P* = 0.56}.

### Strain variation.

A total of 37 strains (types B, E, and F) were captured in eligible studies. The most common strains tested were Eklund 17B (*n* = 70) and Beluga (*n* = 52). The strains for which the *D* value was measured in at least 10 studies (including transformed values) are summarized in Table 3. The highest mean value of *D*′(80) corresponds to strain Saratoga [*D*′(80), ∼2.63 min], whereas the lowest value corresponds to strain Crab 25V-2 [*D*′(80), ∼0.23 min]. The strains with *D* values determined at least 20 times (Eklund 17B, Alaska, ATCC 17786, Beluga, Crab G21-5, Saratoga, and Eklund 202F) were tested for significant differences by one-way ANOVA. Strain Saratoga is significantly more heat resistant than the other strains (*P* < 0.01), whereas strains ATCC 17786 and Crab G21-5 have smaller *D* values. Interestingly, according to statistical analysis, the heat resistance of spores of strains Eklund 17B and Eklund 202F are the same (*P* = 0.3). Cluster analysis reveals no obvious pattern (i.e., all of the strains were identified with a single cluster) based on the heat resistance properties of the strains tested (results not presented).

**TABLE 3:** Heat resistance (*D* values [minutes]) of spores of different strains of nonproteolytic C. botulinum measured at (or transformed to) a heating temperature of 80°C^*a*^

Strain	Toxin type	*n*	<Log[*D*′(80)]>	σ_log[*D*′(80)]_	*n*	<Log[*D*(80)]>	σ_log[*D*(80)]_
Eklund 17B	B	70	0.18	0.49	33	0.35	0.36
Kap B2	B	10	−0.21	0.29	3	0.04	0.37
1304E	E	13	0.09	0.20	3	0.22	0.05
8E	E	14	−0.31	0.39	2	0.08	0.25
Alaska	E	39	0.01	0.45	7	0.28	0.30
ATCC 17786	E	37	−0.21	0.20			
ATCC 9564	E	11	0.40	0.73	5	0.60	0.76
Beluga	E	52	0.10	0.24	12	−0.04	0.19
Crab 25 V-1	E	11	−0.27	0.16			
Crab 25 V-2	E	11	−0.64	0.22			
Crab G21-5	E	27	−0.20	0.37			
Minnesota	E	12	0.02	0.24			
Saratoga	E	47	0.42	0.35	15	0.40	0.28
Craig 610	F	11	0.16	0.28	1	−0.12	
Eklund 202F	F	27	0.14	0.25	1	0.69	
190	F	14	−0.19	0.11			

a *n* is the number of reports, and only strains for which *D* values were reported at least 10 times are included. For strains measured more than once at *T* = 80°C, the mean of the logarithm of reported and transformed values has a correlation coefficient of ∼0.75.

### Effect of heating method.

Analysis of 503 *D* values measured in the absence of lysozyme in a temperature range of 50 to 83°C and transformed to a heating temperature of 80°C reveals that the greatest spore heat resistance was recorded for a single thermal death experiment performed with raw egg white with *D*′(80) = 32.6 min [*D*(83) = 14.2 min, *z* = 6.9] with a type E strain (Alaska). The *D* value was calculated on the basis of a growth/no-growth method by direct incubation of heated medium with injected tryptone peptone glucose yeast extract medium (45). As suggested by others, e.g., Chai and Liang (46), *D* values measured by the TDT method can be higher than those measured by using survival curves. The second highest *D* value was calculated by using data from four experiments conducted with cod fish as a heating menstruum at two temperatures, 75 and 80°C, with strains Eklund 17B (type B) and ATCC 9564 (type E) (47). The weakest resistance corresponds to four experiments performed with 0.03 M phosphate buffer with *D*′(80) = 0.5 min determined for type E strains (8E, 1304E, Minneapolis, and Saratoga) (48). However, when 39 different heating menstrua were divided into two categories assigned to media/buffer (*n* = 258; water, saline, phosphate buffer, and various microbiological growth media) and food matrices [*n* = 245; autoclaved chub fish, béchamel sauce, bolognaise sauce, broccoli puree, carrot homogenates, clam liquor, cod, corn brine, crabmeat, fine carrot, haddock slurry, meat medium, menhaden surimi, milk (evaporated), oyster homogenates, peas, potato puree, precoagulated egg white, raw egg white, salmon, sardines (in tomato sauce), shrimp, tomato homogenates, tuna, tuna (in oil), and whitefish chubs], no significant difference in *D* values was detected (*t* test, *P* = 0.98).

The importance of the heating technique and the method of *D* value determination with respect to the measured spore heat resistance was analyzed by one-way ANOVA. The analysis of *D* values obtained by 11 heating techniques (bottles, flasks, sealed ampoules, sealed capillaries, TDT cans, TDT tubes, unsealed TDT tubes, screw-capped vials and screw-capped tubes in a water bath, and sealed ampoules in an oil bath) indicates a significantly greater mean value of *D*′(80) corresponding to sealed ampules or TDT cans in a water bath (ANOVA, *P* < 0.01). Similarly, across five methods for the measurement of *D* values [labeled survivor curve, TDT, TDT/Stumbo (1948), TDT/Stumbo (1950), and TDT/Stumbo (1957)], TDT and TDT/Stumbo (1948) appear to yield significantly higher *D* values (ANOVA, *P* < 10^−6^). The lowest mean *D* values have been calculated when the spore heat resistance was measured by using a survivor curve.

## DISCUSSION

On the basis of an extensive analysis of previously published information, we have constructed a quantitative representation of beliefs concerning the heat resistance of spores of nonproteolytic C. botulinum. The beliefs are represented by parameters that describe probability distributions for *z* values and for *D* values at a fixed temperature of 80°C (these parameters describe linear kinetics of thermal inactivation over a range of operating temperatures). A distinct set of parameters corresponds to information that relates to the heat resistance of spores recovered in the presence of lysozyme. The probability distributions dominantly represent uncertainty in relation to the parameter values but also include some elements of variability associated with distinct bacterial strains and experimental methods. The parameterized form facilitates the inclusion of information about the heat resistance of spores into quantitative risk assessment for food-borne botulism hazards; this is an essential element in the safety management of chilled food products. The detailed statistical representation of both *D* values and *z* values ensures that the relevant uncertainties can be combined correctly to establish an uncertain *D* value at other temperatures.

**FIG 4:**
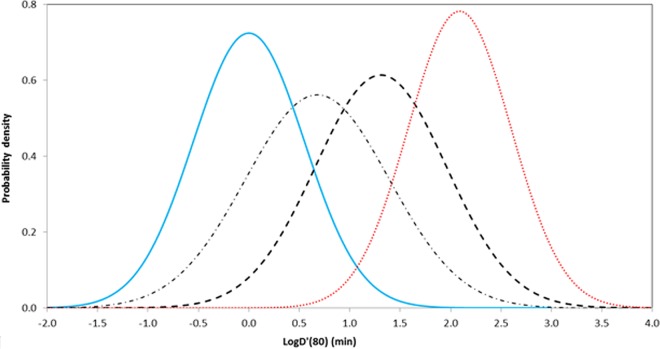
Distribution of log[*D*′(80)] for spores of nonproteolytic C. botulinum. The blue solid line represents beliefs from the present study, based on a large collection of previously published results, for the heat resistance of spores in the absence of lysozyme [<log*D*′(80)> = 0.00, σ_log[*D*′(80)]_ = 0.55, <*z*> = 6.7], and the red dotted line represents beliefs for inactivation of the HR fraction of spores in the presence of lysozyme [<log*D*′(80)> = 2.09, σ_log[*D*′(80)]_ = 0.51, <*z*> = 9.3]. The dotted-dashed line illustrates a probability distribution for *D* values estimated by van Asselt and Zwietering (26) [<log*D*′(80)> = 0.68, σ_log[*D*′(80)]_ = 0.71, <*z*> = 18.6], and the dashed line represents a distribution based on the guidance of the United Kingdom Food Standards Agency of 90°C for 10 min (49) [<log*D*′(80)> = 1.31, σ_log[*D*′(80)]_ = 0.65, <*z*> = 9.2; with the coefficient of variation of the logarithm of the *D* value taken as 0.5].

Probabilities concerning the transformed *D*′(80) values converted from data established over a range of heating temperatures are compared with two other representations in Fig. 4. The results of van Asselt and Zwietering (26) fall between the values associated with heat resistance measured with recovery in the presence or absence of lysozyme. Note that van Asselt and Zwietering did not segment their data with respect to lysozyme and that their chosen reference temperature (*D*_ref_ = 120°C) is considerably higher than the majority of the operating temperatures that are associated with nonproteolytic C. botulinum hazards. The distribution constructed to represent the guidance given by the United Kingdom Food Standards Agency (49) covers higher (more conservative) *D* values than those observed for recovery in the absence of lysozyme but does not consider recovery in the presence of lysozyme explicitly (Fig. 4).

In addition to the separation of systems that involve lysozyme (Fig. 4), another important development was to exclude thermal data collected at temperatures above 83°C (when spores were recovered in the absence of lysozyme) (see above). The implication of this development was that the real *D* values were lower than those reported at higher heating temperatures. The origins of distinctions for *D* values that correspond to the higher operating temperature are unclear. Decimal reduction times at higher temperatures (∼90°C) have values in the range of 0.01 to 1 min, so that actual physical measurement may present problems. Alternatively, there may be real changes in heat resistance as the physical properties of spores (particularly the transport properties of the spore coat) change in response to higher temperatures.

Experiments involving lysozyme indicate that the heat-resistant fraction usually represents 0.02 to 3.1% of the spore population. This agrees with previous estimates and reflects the fraction of spores with coats that are permeable to lysozyme (37). This includes both situations where lysozyme is added deliberately to the recovery medium and those in which it is present in the substrate used for recovery, e.g., vegetable juices or crabmeat (50, 51). In a study by Peck et al. (35), a biphasic survivor curve, and consequently the increased number of surviving spores, was observed with a lysozyme concentration of 0.1 μg ml^−1^ and the maximum spore recovery corresponded to a concentration of ∼5 to 10 μg ml^−1^. Significantly higher *D* values, and *z* values, in systems with lysozyme indicate that this property of the substrate is an important variable in risk assessments. Lysozyme has been reported in many types of raw foods and may survive the heat treatments typically applied to chilled foods (31, 33, 35–39, 41, 51–55).

Studies of the genomic variability of nonproteolytic C. botulinum have revealed that strains forming type B and F toxins are closely related and distinct from most type E toxin-forming strains (8, 9, 14, 56–59). Physiological differences have also been described, for example, in different trends of carbohydrate utilization, with type B and F strains producing acid from amylopectin, amylose, and glycogen but not from melezitose or inositol and type E strains showing the opposite trend (14). However, although there was strain variability, there did not appear to be a clear relationship between the toxin type and the minimum growth temperature or the maximum NaCl concentration permitting growth (14). In the present study, statistical tests indicate that there is only a weak relationship between the toxin type and spore heat resistance, although some authors have reported the measured spore heat resistance of type B strains to be greater than that of type E strains (33, 37, 60). In the present study, of the seven strains (one type B, five type E, and one type F) that had been studied more than 20 times, strain Saratoga (a type E strain) had the highest measured spore heat resistance. The generation of thermal death data from a significant number of strains in a reproducible way would be of great value.

**FIG 5:**
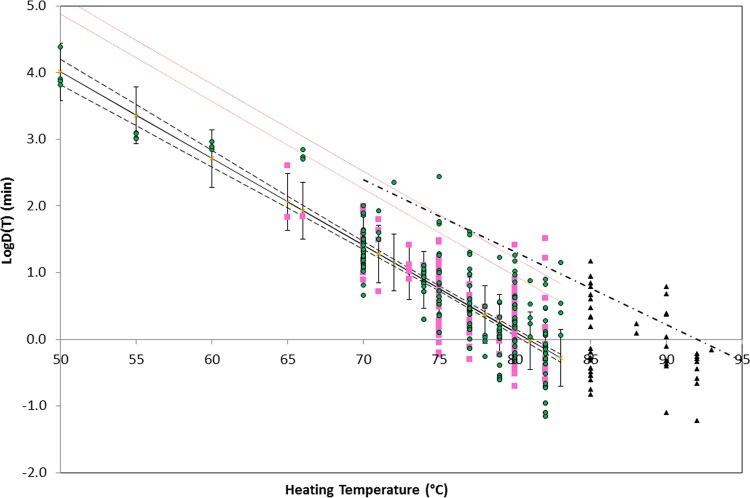
Logarithms of previously published *D* values and fitted models of the heat resistance of spores of nonproteolytic C. botulinum in foods (green circles) and laboratory media (pink squares) that do not contain lysozyme. Black triangles indicate data that correspond to heating at temperatures that exceed 83°C, which are not included in the model. The black solid line is the line of best fit {log[*D*(*T*)] = 10.506 − 0.1299*T* with *r* = 0.72}, and the dashed lines indicate the 95% confidence interval for the fit. Red dotted lines represent the 95% and 99% UCLs of the predicted response. The dashed-dotted line indicates the current guidance of the United Kingdom Food Standards Agency (49)/Advisory Committee on the Microbiological Safety of Food (66) for heat treatments applied to chilled foods. Data are from references 31, 33–37, 40, 41, 45–48, 50, 51, 55, 60, 62–65, and 67–90 and unpublished work by J.-M. Membré and P. McClure and at the Institute of Food Research.

The *D* values collected in this report have a direct application in risk management. On the basis of the 99% upper confidence limit (UCL) of predicted *D* values (in the absence of lysozyme and for heating temperatures below 83°C), the time required to reduce the spore concentration by a factor of 10^6^ at 90°C is ∼5 min (Fig. 5). This value indicates that the current guidance of the United Kingdom Food Standards Agency (49) and the Chilled Food Association (61) (Table 4) provides a suitable level of safety. Six *D* values, from five different studies, have values larger than that the 99% UCL for the predicted response. Two, from Scott and Bernard (60), who reported *D*(82) = 32.3 min and *D*(82) = 16.7 min, correspond to heating in 0.067 M phosphate buffer. Bohrer et al. (62) reported *D*(72) = 226 min for spores heated in tuna (in oil), and Alderman et al. (45) reported *D*(83) = 14.2 min for raw egg white. *D*(80) = 25.8 min can be calculated from data reported by Murrell and Scott (63), but the heating menstruum and incubation time were not reported and *D*(75) = 275 min can be calculated from studies by Fernández and Peck (64). Four of these *D* values were obtained following prolonged incubation (90 to 336 days) (60, 62, 64). It has been observed by several authors, e.g., by Lynt et al. (65), that an extended period of incubation permits germination of damaged spores and so increases the measured spore heat resistance. Such observations are important when translating relative short-term experiments into long-term shelf lives of chilled foods. Four of these values are based on the TDT method, and the actual population inactivation kinetics were not reported. The *z* values collected in this report have a direct application in risk management. A suitable *z* value of 6.7°C is indicated from the analysis of the literature data (for recovery in the absence of lysozyme) and is consistent with the *z* value of 7°C advocated by the Chilled Food Association, compared to the *z* value of 9.2°C advocated by the United Kingdom Food Standards Agency (49) (Table 4); analysis of the reported *D* values (Fig. 5) indicates a *z* value of 7.7°C, in the middle of this range.

**TABLE 4:** Heating time required to reduce the concentration of nonproteolytic C. botulinum spores by a factor of 10^6^^*a*^

*T* (°C)	Time (min)				
	UKFSA 90 for 10 (*z* = 9.2°C)	CFA 90 for 10 (*z* = 7°C)	Model from current literature review		
			Expected value	95% UCL	99% UCL
70	1,675		155	1,092	2,019
75	464		35	243	448
80	129	270	7.8	54	100
85	36	52	1.8	12	22
90	10	10	0.4	2.7	4.9

a According to the United Kingdom Food Standards Agency (UKFSA) (49), the Advisory Committee on the Microbiological Safety of Food (66), the Chilled Food Association (CFA) (61), and a model developed from previously published literature (for data obtained without lysozyme in the recovery media).

### Conclusions.

A large data set of previously published *D* values for nonproteolytic C. botulinum spore inactivation during heating provides evidence for the development of mathematical models that allow general conclusions regarding heat resistance and support quantitative risk assessment for food-borne hazards. The models express current information uncertainty, and associated analysis points to possible origins of population variability.

This review indicates that it is crucial to take care when interpreting information regarding the thermal inactivation of spores of nonproteolytic C. botulinum because experiments that involve lysozyme or heating temperatures of >83°C require independent analyses. In addition, it is important to appreciate that the models developed in this study describe the reduction of spore population size for nonproteolytic C. botulinum following heating but do not quantify the properties of survivors, such as the ability to germinate and grow, that may be relevant for risk assessment.

Current advice does not consider the influence of lysozyme present in heated food products. This review, besides describing parameter values that can be used in QMRA, is strong evidence for separate treatments of foods that include lytic enzymes. On the basis of the results of this review (using data from Table 2 and the estimate of the heat-resistant fraction), heated spores recovered in the presence of lysozyme may require ∼80 min at 90°C to reduces the spore concentration by a factor of 10^6^.

## Supplementary Material

Supplemental material
