# Supplementary material for: Systematic Assessment of Nonproteolytic Clostridium botulinum Spores for Heat Resistance
Source: Appl Environ Microbiol. 2016 Sep 16;82(19):6019–29. doi: 10.1128/AEM.01737-16 (PMC5038052; doi:10.1128/AEM.01737-16)
Supplement: Supplemental material [file supp_82_19_6019__index.html]

Supplemental material 

# Systematic Assessment of Nonproteolytic Clostridium botulinum Spores for Heat Resistance

## Supplemental material

- Supplemental file 1 -

  Data set of obtained *D* and *z* values.

  XLSX, 105K
